# Association Study of Genes Associated to Asthma in a Specific Environment, in an Asthma Familial Collection Located in a Rural Area Influenced by Different Industries

**DOI:** 10.3390/ijerph9082620

**Published:** 2012-07-27

**Authors:** Andréanne Morin, Jeffrey R. Brook, Caroline Duchaine, Catherine Laprise

**Affiliations:** 1 Université du Québec à Chicoutimi, 555 boulevard de l’Université, Saguenay, QC G7H 2B1, Canada; Email: andreanne1_morin@uqac.ca; 2 Air Quality Processes Research Section, Environment Canada Dalla Lana School of Public Health, University of Toronto, 4905 Dufferin St., Toronto, ON M3H 5T4, Canada; Email: Jeff.Brook@ec.gc.ca; 3 Institut Universitaire de Cardiologie et Pneumologie de Québec, Hôpital Laval 2725, Chemin Sainte-Foy, QC G1V 4G5, Canada; Email: Caroline.Duchaine@bcm.ulaval.ca; 4 Community Genomic Medicine Centre, University of Montréal, Chicoutimi University Hospital, 305, Rue Saint-Vallier, C.P. 5006, Saguenay, QC G7H 5H6, Canada

**Keywords:** asthma, gene-environment interactions, aluminium industries, pulp and paper industries, air pollution

## Abstract

Eight candidate genes selected in this study were previously associated with gene-environment interactions in asthma in an urban area. These genes were analyzed in a familial collection from a founder and remote population (Saguenay–Lac-Saint-Jean; SLSJ) located in an area with low air levels of ozone but with localized areas of relatively high air pollutant levels, such as sulphur dioxide, when compared to many urban areas. Polymorphisms (SNPs) were extracted from the genome-wide association study (GWAS) performed on the SLSJ familial collection. A transmission disequilibrium test (TDT) was performed using the entire family sample (1,428 individuals in 254 nuclear families). Stratification according to the proximity of aluminium, pulp and paper industries was also analyzed. Two genes were associated with asthma in the entire sample before correction (*CAT* and *NQO1*) and one was associated after correction for multiple analyses (*CAT*). Two genes were associated when subjects were stratified according to the proximity of aluminium industries (*CAT* and *NQO1*) and one according to the proximity of pulp and paper industries (*GSTP1*). However, none of them resisted correction for multiple analyses. Given that the spatial pattern of environmental exposures can be complex and inadequately represented by a few stationary monitors and that exposures can also come from sources other than the standard outdoor air pollution (e.g., indoor air, occupation, residential wood smoke), a new approach and new tools are required to measure specific and individual pollutant exposures in order to estimate the real impact of gene-environment interactions on respiratory health.

## 1. Introduction

Asthma is a chronic inflammatory disease of the airways characterized by airway hyperresponsiveness, inflammation and remodelling. Asthma is a complex trait and many risk factors have been identified. These risk factors can be divided into two main categories: genetics and environmental factors [1]. The mechanisms underlying the implication of these two categories in the development and the expression of asthma have not been completely clarified. However, it has been found that the interactions between genetic and environmental factors can induce the expression of the disease phenotype [2]. Studies evaluating the impact of ambient air pollution in the development of the disease have shown growing evidence that exposure to ambient air pollution can lead to the development of the asthma phenotype [2].

As reviewed by London and Romieu in 2009 [2], different genes associated with the disease phenotype in interaction with air pollution have been identified in the past few years. These studies have contributed to the understanding of the pathophysiology of asthma and to the comprehension of how different environments can have a protective or deleterious effect on the disease. In the literature, different categories of genes were associated with asthma in populations exposed to a polluted urban environment. The principal categories reported are immune signalling (mediators) molecules such as: tumor necrosis factor (*TNF*) and transforming growth factor, beta 1 (*TGFB1*) [3,4,5,6]; immune response molecule: myeloperoxidase (*MPO*); free radical metabolism molecules: catalase (*CAT*), epoxide hydrolase 1, microsomal (*EPHX1*), glutathione S-transferase pi 1 (*GSTP1*) and NAD(P)H dehydrogenase, quinone 1 (*NQO1*) [7,8,9,10,11,12,13,14,15] that protect against reactive oxygen species (ROS) produced by air pollutants [16]; and metabolic enzyme like arginase (*ARG1*). 

The objective of this study was to evaluate the association between asthma-related phenotypes and eight genes selected from the literature based on their interaction with asthma in an environment characterized by a higher level of air pollution. The analyses were performed on a familial asthma collection located in the Saguenay–Lac-Saint-Jean region (SLSJ)—a region located in the northeastern Quebec province of Canada. This sample is a distinct, remote population, which is exposed to different levels of air pollutants compared to urban populations. Many industries influence the levels of air pollutants of this region: industries associated with aluminium, pulp and paper. Wood products industries are also present. 

## 2. Methods

### 2.1. Subjects

A well-characterized family sample of asthmatic patients from the founder population of SLSJ was used to investigate the association between genes selected based on interaction reported with specific environmental exposure and asthma. Asthma phenotype was described following the American Thoracic Society standards [17]: all participants had a respiratory health questionnaire and function tests. Participants were defined as having asthma if (1) they had a reported history of asthma (validated by a physician); or (2) they showed asthma-related symptoms and a positive PC_20_ at the time of recruitment. Subjects with a PC_20_ greater than 8 mg/mL; without history of physician-diagnosed asthma, and without symptoms of asthma; and with no positive response on skin prick test were considered unaffected for asthma. Detailed recruitment method is described in the paper by Bégin *et al.* 2007 [18]. Probands were included in the study if they met two of the three following criteria: (1) a minimum of three clinic visits for acute asthma within one year; (2) two or more asthma related hospital admissions within one year; (3) steroid dependency, defined by either a use of oral corticosteroids for six months, or a year for inhaled corticosteroids. Families were included in the study if phenotypic assessment was available for at least one parent, at least one parent was unaffected, and if all grandparents were French-Canadian descendants. Family members were considered asthmatics if both a self-reported history of asthma and a history of physician diagnosed asthma were recorded, or by clinical evaluation following a methacholine provocation test. Spirometry, methacholine challenge and IgE measurements are detailed in Bégin *et al.* 2007 [18]. Spirometry was performed to measure the expiratory flow (FEV_1_) using a Morgan spirometer (Morgan Spiro 232, P.K. Morgan Ltd.) following the American Thoracic Society’s recommendations [19]. Methacholine challenges (bronchoprovocation) were performed for participants of 12 years and older according to the method described by Juniper *et al*. [20]. The PC_20_ is described as the dose of methacholine that resulted in a 20% fall in FEV_1_. Serum IgE was measured with enzyme immunofluorometry. The investigators performed all of the measures for each participant (except for bronchoprovocation) at the University of Montreal Community Genomic Medicine Centre in Saguenay, Québec, Canada. All subjects signed the informed consent approved by the local hospital (Centre de santé et de service sociaux de Chicoutimi) ethics committee. A total of 1,428 individuals from 254 nuclear families were included the study (Table 1).

### 2.2. Gene Selection and Genotypes

Eight genes were selected from the literature: immune signalling molecules (*TGFB1* and *TNF*), immune response molecule (*MPO*), genes implicated in free radical metabolism (*CAT*, *EPHX1*, *GSTP1* and *NQO1*) and metabolic enzyme (*ARG1*). The specific genes were selected in the literature using PubMed. Genes had to have one or more polymorphisms (SNPs) associated to asthma in an environment related to air pollution (pollutant related to industries or other pollutant found in the SLSJ region). Key words used were: air pollution, carbon monoxide, ozone, nitrogen oxide, particulate matter, sulfur dioxide and industries. 

**Table 1:** Clinical characteristics of the Saguenay-Lac-Saint-Jean asthma study.

	All samples (n = 1,428)	Probands ^a^ (n = 254)	Family members (n = 1,174)	Exposed ^b^	
				Children (n_a_ = 327) (n_p_ = 194)	Family members (n_a_ = 729)(n_p_ = 431)
**General descriptive data**					
M:F ratio	1:1.2	1:1.2	1:1.2	1:1.2	1:1.1
				1:1.4	1:1.2
Age, mean (range) ^c^	39 (2–93)	18 (3–62)	43 (2–93)	22 (2–71)	47 (3–93)
				25 (2–75)	47 (3–88)
Age median	39	16	43	17	45
				19	45
Smoking status, n (%) ^d^					
non smoker	721 (51)	208 (84)	512 (44)	239 (74)	279 (39)
				139 (74)	163 (39)
ex smoker	405 (29)	14 (6)	391 (34)	38(12)	277 (39)
				22 (12)	157 (37)
smoker	283 (20)	27 (11)	256 (22)	47 (14)	162 (22)
				27 (14)	103 (24)
**Clinical descriptive data**					
FEV_1_, % pred. (SD) ^e^	94 (42)	93 (34)	95 (43)	93 (18)	96 (18)
				91(16)	95 (18)
PC_20_, mg/mL (SD) ^f^	7.76 (5.21)	2.50 (3.61)	10.06 (5.04)	6.40 (10.85)	27.83 (26.4)
				7.60 (13.02)	29.30 (26.98)
IgE, μg/L (SD) ^g^	135 (5)	236 (5)	118 (4)	693 (1738)	260 (517.1)
				891 (3063.9)	394 (1975.6)
Asthma, n (%) ^h^	693 (49)	253 (100)	440 (38)	327 (100)	192 (27)
				194 (100)	116 (27)

^a^ Probands are first affected family member recruited in the familial collection and family members refers to other family members who joined the study; ^b^ Exposed refers to the stratification according to the proximity of aluminium (n_a_ (upper line)) and pulp and paper (n_p_ (lower line)) industries. A total of 382 individuals (168 children) are exposed to both aluminium and pulp and paper industries; ^c^ Mean and median age calculated for 1,425 subjects (254 probands and 1,172 family members); ^d^ Smoking status available for 1409 subjects (250 probands and 1159 family members) and passive smoking available for 1,240 subjects (239 probands and 1001 family members). Ex smoker are defined as subjects who have stopped smoking 3 months or more; ^e^ FEV_1_ = Mean and standard deviation (SD) calculated for the Forced expiratory volume in one second in % of predicted value for 1133 subjects (221 probands and 912 family members); ^f^ PC_20_ = Provocative concentration of methacholine that induces a 20% fall in FEV_1_. Geometric mean and SD calculated for 1,045 subjects (196 probands and 849 family members); ^g^ IgE = Immunoglobulin E serum concentration. Geometric mean and SD; ^h^ Present asthma or past documented clinical history of asthma. The reported mean age of onset is 7 years old among probands and 22 years old among asthmatic family members. Asthma phenotype is available for 1,166 of the 1,174 family members.

SNPs were extracted using the PLINK software from the genome-wide association study (GWAS) performed on the SLSJ familial collection in the context of the large-scale, consortium-based genome wide association study of asthma GABRIEL [21]. SNPs extracted were located in the selected genes and their 5’ and 3’ untranslated regions (UTR) (which may contain gene expression regulation sites and promoter regions). A total of 90 SNPs were extracted and a total of 72 were kept for analysis (criteria are mentioned in the statistical analysis section): 64 in the total sample, 69 when stratified according to proximity of aluminium industries and 69 when stratified according to pulp and paper industries. All SNPs are described in Table 1 in the supplementary material.

### 2.3. Environment Characteristics and Air Pollution Data

Subjects of the SLSJ sample were recruited from 1998 to 2001. During these years, only outdoor ozone and sulphur dioxide concentrations were measured routinely in four different places in the SLSJ area. Table 2 is a brief summary of the measures for ozone and sulphur dioxide among these four sites between 1997 and 2000. Both of these pollutants are known to pose a risk to human health and thus ambient air quality standards are in place provincially, federally and also in other countries [22,23]. Air pollutant measures are in part per billion (ppb) and are assessed during a defined period of time (8 hours or 24 hours for longer periods and 1 h for peaks). For example, standards set by the *Ministère du Développement durable, de l’Environnement et des Parcs*, indicate that the acceptable limits are a maximum value of ozone of 65 ppb for 8 hours and/or 80 ppb for 1 h. The maximum acceptable limits for sulphur dioxide (SO_2_) are set at 20 ppb for an annual average, 110 ppb for a 24 h average and 500 ppb for 1 h. The levels observed in U.S. cities exceed the standards: the average range of ozone is 0 to 125 ppb for 8 h and the average for SO_2_ is 0.5 to 50 ppb for 24 h. Also peaks of 200 ppb for 1 h of ozone and 150 ppb for 1 h of SO_2_ are typically observed in U.S. cities that are not located in areas where there are direct emission sources [24].

As shown in Table 2, ozone and SO_2_ levels cannot be considered low in SLSJ region because the concentrations are close to the limit value set by the Canadian government and to levels measured in more populated areas of Canada, as well as in the United States [24]. In particular, the magnitude of the SO_2_ peaks occurring indicates that the industries known to operate in the area have a significant impact on air quality. Additionally, they are potentially leading to at least some exposure for the study population depending upon the prevailing winds. 

The majority of the subjects live within a 10 km radius of aluminium industries (73.93%), which are known to emit significant amounts of fluoride, polycyclic aromatic hydrocarbons (PAH), SO_2_, carbon monoxide (CO) and particulate matter (PM_2.5_ and PM_10_) (NPRI Google Earth tool) (Table 2, supplementary material). There is also a good proportion of the individuals living near pulp and paper industries (44.59%) and wood product industries (11.80%). Such facilities are known to emit a range of air pollutants, such as volatile organic compound (VOC), PM_2.5_ and PM_10_, sulphur oxides (SO_x_), and nitrogen oxides (NO_x_) *etc*. [26,27]. There was also a small proportion of the individuals living within a 10 km radius of a niobium mine and other industries, including milk transformation, iron and steel, petroleum refining, chemicals, plastic and rubber *etc*. Air pollution emissions in tones during the year 2002 are indicated in Table 3 in the supplementary material for each industry located in the SLSJ area.

**Table 2:** Levels of air pollutants measured in four places in the SLSJ area.

Air pollutant	Years	Mean annual value (ppb)	Period (hour) ^a^	Median (ppb)	Maximum (ppb)
**Ozone**	1997 to 2000	27.7	1	28	76.2
			8	27.8	72.2
**Sulphur dioxide**		9.41	1	1.6	150
			24	3.7	82.4

^a^ Periods are defined as: 1 h = mean value hourly, 8 h = mean value during 8 hours and 24 h = mean value during 24 hours. Areas where sulphur dioxide was measured were influenced by different industries [25].

### 2.4. Statistical Analysis

SNPs were tested for association with asthma using the transmission disequilibrium test (TDT) performed with PLINK analysis software [28,29]. The association study was performed on a familial collection. Thus, to test if SNPs are associated to the trait, transmission disequilibrium is observed between generations. Differential allele transmission to affected offspring from heterozygous parents was calculated to determine the association. SNPs included in the study must fulfill these quality criteria: a cut off for minor allele frequency of 5%, a genotyping rate of 5%, a *p* value > 0.05 for the Hardy-Weinberg equilibrium and < 1% of Mendelian errors. TDT analyses were performed for SNPs and haplotypes employing a chi-square distribution followed by permutations to determine the significance. Permutations were used to test the accuracy of the estimated *p* value and to palliate lack of power and false positive results. The number of permutations was chosen according to the significance level of the *p* values of the TDT analyses (for example: 10,000 permutations for *p* < 0.05 and 20,000 permutations for *p* < 0.01). Haplotypes (combination of alleles transmitted together) and strength of linkage disequilibrium between SNPs (the more these alleles are transmitted together, the more they are dependent and thus in linkage disequilibrium, values shown as D’) were obtained using the Haploview software (version 3.31; Broad Institute of MIT and Harvard University, Boston, MA, USA). Association between different haplotype blocks of SNPs and asthma was also done using the TDT analysis in the PLINK software. According to the number of SNPs considered in the TDT analyses, multiple analyses correction was performed to avoid false positive results. This correction was done considering the number of independent tagSNPs (SNPs that represent a region with high linkage disequilibrium) for each gene separately using the Nyholt method [30]. For example, for *CAT*, there were 11 independent tagSNPs and the corrected threshold was 0.0047 (0.05/11 tagSNPs). 

TDT for SNPs and haplotype blocks were repeated after stratification for the proximity of aluminium or pulp and paper industries (10 km radius around the subject’s house location). For these analyses, only the nuclear trios, for which the children were exposed to one industry or the other, were kept for analyses. 

## 3. Results

Association results for SNPs that had a significant *p*-value before correction (*p* < 0.05) for the whole sample and for the sample stratified according to the proximity of aluminium or pulp and paper industries are shown in Table 3. Five SNPs from two genes (*CAT* (rs11032703, rs2300181, rs511895) and *NQO1* (rs1800566, rs1437135)) were associated before correction and one SNP located in *CAT* gene was still associated after correction (indicated in bold in Table 3). The minor allele of the *CAT* rs11032703T SNP was transmitted more often to asthmatic subjects and could be a risk factor of asthma. Results for the TDT with the stratified sample that had a *p*-value less than the 0.05 thresholds are indicated in Table 3. Three SNPs located in two genes were associated when stratified according to proximity to aluminium industries (*CAT* (rs1132703) and *NQO1* (rs1800566 and rs1437135)) and only one SNP in the *GSTP1* gene (rs1695) was associated in the pulp and paper sample. None of the SNPs resisted the correction for multiple analyses.

Haplotypes of the selected genes were also analyzed to look for association with asthma in the whole sample and the stratified ones. A TDT was then performed on linkage disequilibrium plot located in the eight genes selected for this study and obtained with the Haploview software. Five haplotypes located in three different genes (*CAT*, *EPHX1* and *NQO1*) were associated to asthma before correction (*p* < 0.05) and are shown in Table 4. One haplotype located in the *CAT* gene was still associated after correction. The haplotype was transmitted more often to asthmatic patients, which indicates a potential deleterious effect on the phenotype. This haplotype also contains the SNP rs11032703 that has been associated alone with asthma, but also other SNPs that had a *p* value under 0.05, but did not remain significant after correction (rs2300181 (intron 6) and rs511895 (intron 10)). Linkage disequilibrium plot for the *CAT* gene is shown in Figure S1 in the supplementary materials. Two haplotypes located in two genes (*CAT* and *NQO1*) were associated to asthma before correction when the sample was stratified according to proximity to aluminium industries. When stratified according to proximity to pulp and paper industries there were also two haplotype blocks located in two genes (*EPHX1* and *GSTP1*) that were associated to asthma before correction. None of these haplotypes resisted correction for multiple analyses.

**Table 3:** Transmission disequilibrium test (TDT) results with *p* value < 0.05 for genes selected in the literature and stratified according to proximity to aluminium or pulp and paper industries.

Industry	Gene symbol/position	SNP/position ^a^	Allele ^b^	T	U	OR	CHISQ	*p* value ^a^	*p* value with permutations	*p* value threshold (Meff) ^c^
**All samples**	*CAT/*11p13	**rs11032703/intron 1**	T/**C**	90	52	1.731	10.17	0.001428	**0.0029**	0.00474
		rs2300181/intron 6	T/**C**	90	120	0.75	4.286	0.03843	0.04225	(10.5501)
		rs511895/intron 10	T/**C**	170	129	1.318	5.622	0.0177	0.0227	
	*NQO1*/16q22.1	rs1800566/exon 5 (Pro187Ser)	A/**G**	86	118	0.7288	5.02	0.02506	0.03135	0.01686
										(2.9652)
		rs1437135/intron 5	G/**A**	86	118	0.7288	5.02	0.02506	0.03135	
**Aluminium**	*CAT/*11p13	rs11032703/intron 1	T/**C**	71	41	1.732	8.036	0.004586	0.0055	0.00442
										(11.3167)
	*NQO1*/16q22.1	rs1800566/exon 5 (Pro187Ser)	A/**G**	63	90	0.7	4.765	0.02905	0.03595	0.01827
										(2.7369)
		rs1437135/intron 5	G/**A**	63	90	0.7	4.765	0.02905	0.03595	
**Pulp and Paper**	*GSTP1/*11q13	rs1695/exon 5 (Ile105Val)	**G**/A	46	70	0.6571	4.966	0.02586	0.0354	0.02072
										(2.4134)

Definition of abbreviations: SNP= single nucleotide polymorphism, T = Transmitted minor allele count, U = Untransmitted allele count, OR = odds ratio, CHISQ = chi-square statistic, *CAT* = catalase, *GSTP1* = Glutathione S-transferase, *NQO1* = NAD(P)H dehydrogenase quinone 1; ^a^ Significant results after corrections are indicated in bold; ^b^ Ancestral alleles are indicated in bold when available in National Centre for Biotechnology Information (NCBI); ^c^ Corrected *p* value threshold for each gene considering only tagSNPs [30]. Meff = Effective number of independent marker loci.

**Table 4:** Transmission Disequilibrium Test (TDT) results with *p* value < 0.05 for haplotypes and stratified according to the proximity of aluminium of pulp and paper industries.

Industry	Gene symbol/position	Locus	Haplotype	T	U	CHISQ	*p* value ^a^	*p* value Threshold ^b^(Meff)
All samples	*CAT*/11p13	H1	rs208679A rs7118388G rs7944397A rs208682C rs554518C	162.5	123.8	5.223	0.0223	0.00474 (10.5509)
		H1	rs208679A rs7118388A rs7944397A rs208682C rs554518C	113.5	148.9	4.779	0.0288	
		H2	**rs1001179C rs480575A rs2284369A rs11032703T rs769218G rs2300181C rs7933285C rs511895T rs10488736C**	91	54.4	9.221	**0.002406**	
	*EPHX1*/1q42.1	H3	rs4653695A rs2740174A rs360063A rs1009668C	150.8	114.9	4.842	0.02777	0.00352 (14.2206)
	*NQO1/*16q22.1	H1	rs1800566A rs4986998G rs689452G rs1437135G rs689457C rs2917682T	86	119	5.312	0.02118	0.01686 (2.9652)
Aluminium	*CAT*/11p13	H2	rs1001179C rs480575A rs2284369A rs11032703T rs769218G rs2300181C rs7933285C rs511895T rs10488736C	71	42	7.442	0.00637	0.00442 (11.3167)
	*NQO1/*16q22.1	H1	rs1800566A rs4986998G rs689452G rs1437135G rs689457C rs2917682T	63	91	5.091	0.02405	0.01827 (2.7369)
Pulp and Paper	*EPHX1*/1q42.1	H4	rs360063A rs1009668C	62	41	4.282	0.03853	0.00311
	*GSTP1*/ 11q13	H1	rs1695A rs1138272C	69	45	5.053	0.02459	0.02072 (2.4134)

Haplotypes were selected from all the genes selected for this study. Definition of abbreviations: T = transmitted minor allele count, U = untransmitted allele count, *NQO1* = NAD(P)H dehydrogenase quinone 1, *CAT* = catalase, *EPHX1* = epoxide hydrolase 1, microsomal, CHISQ = chi-square statistic; ^a^ Significant results after correction; ^b^ Corrected *p* value considering the number of independent tagSNPs [30]. Meff = Effective number of independent marker loci.

## 4. Discussion

We performed a genetic family based association study between asthma and genes that were previously associated with air pollution in an urban environment. Our study focused on a population residing in a relatively unpopulated area but where the air quality is influenced by the presence of several industries. The principal strength of this study is the well-defined population. Every subject has gone through different analyses of their respiratory capacity and they were questioned on different aspects related to respiratory diseases like their smoking status. It is also a homogeneous population with similar lifestyles (same religion, language, activities, *etc*.) [31].

This study also has some limitations. First, the familial collection was not designed to study gene-environment interactions. The main purpose is to study the impact of genetic variants on asthma phenotypes.

Also, the presence of a significant number of smokers (11% of probands and 20% of family members) or ex-smokers (6% of probands and 29% of family members) might have influenced the results of the gene-environment interactions examined in this study. For example, the two associated genes (*CAT* and *NQO1*) have been associated with asthma and tobacco smoke exposures [32,33]. *NQO1* Pro187Ser mutation was associated in our study, but was also associated with asthma and exposure to tobacco smoke in a previous study. Unfortunately, excluding smokers in this study would have significantly affected its power. 

Potential environmental exposures for this study were complex and difficult to describe given the lack of information on true spatial concentration patterns and subject activity. Thus, while it would have been interesting to consider exposure of each individual, such detail was beyond the scope of the present study. Compounding these challenges, the concentration of some key air pollutants, like PM_10_, PM_2.5_, PAH, CO and VOC, were not measured in the area during the recruitment years. However, as described above data on total release of different pollutants were available for the year 2002. Furthermore, even if data were not available during the recruitment years (1998 to 2001), the 2002 data are still relevant as an indication of the general air conditions in the area. This is because the air pollutants emitted by the different industries generally do not show significant differences from one year to the next. Decreasing trends are only apparent over multiple years.

Even if the distances between the industries and the homes of the subjects were measured and the dispersion of the emitted pollutants were known so that more accurate air pollutant exposures could be assigned there are other potential exposures in this population. For example, occupational exposures or local activities can be significant, but with current information these cannot be quantified. There are also other types of oxidizing pollutants like indoor air exposures (e.g., environmental tobacco smoke) or wood smoke from residential burning that can influence individual exposures and these are also not known for our population. Many contaminant from wood smoke can have an impact on health like CO, PM_2,5_, NO_x_, PAH, *etc*. [34]. 

Another limitation with this familial collection is that some of the genes were not evaluated because few SNPs located in these genes were not analyzed in the ILLUMINA 610K arrays or these SNPs did not respond to criteria mentioned in the statistical analysis section. This was the case for the *MPO* gene (rs2333227) that was previously associated with asthma in gene-environment interactions studies [15]. 

Candidate gene studies have a low replication rate [35], that can be explained by the difference in recruitment (case-control studies compared to familial studies or birth cohorts, different age of onset in cohorts, *etc*.), by the presence of difference in linkage disequilibrium between populations (a founder population compared to a cosmopolitan population) and in the characterization of the subjects at the phenotypic and exposition to environment levels. In this study, we tried to reduce the impact of the difference in the characterization of exposure to the environment by selecting genes that were previously associated to asthma in similar environment (urban area with pollutant related to industries or other pollutant found in the SLSJ region). Gene selection was well defined and based on their previous association with asthma and air pollution in the literature and criteria are mentioned in the Gene Selection and Genotypes section. This strict selection allowed replicating selected genes in a precise environment to address the impact of their interaction with this environment in the context of asthma pathogenesis. 

This study showed an association for the *CAT* gene and asthma in the whole sample of the SLSJ study. The *CAT* gene is located on the chromosome 11p13 and expresses the catalase protein implicated in defense against oxidative stress [36]. It is an antioxidant enzyme that protects the cell from oxidative stress by decomposition of hydrogen peroxide into water and oxygen [36]. Reactive oxygen species and hydrogen peroxide are known to have an impact on many characteristic elements of the asthma phenotype such as contracting airway smooth muscle, increasing airway reactivity and the synthesis and release of chemoattractants [36,37,38,39]. There are different external and cellular sources of hydrogen peroxide like ozone or inflammatory cells and the respiratory chain located in the mitochondria [36]. Catalase may play a role on the oxidant-antioxidant imbalance in asthma because studies have shown a higher activity of the enzyme in affected people [40].

Our results show an association between *CAT* rs11032703 and asthma in the SLSJ sample and show that the minor allele might be a risk factor for the disease phenotype. Two studies have previously associated this gene with exposure to air pollutants [15,41]. Wenten *et al.* found an epistasic effect between SNPs in *CAT* (rs1001179) and *MPO* (rs2333227) genes when subjects were exposed to air pollutants (NO_2_ and ozone) [15] but neither of the two SNPs alone was associated with the phenotype combined with the environment. Strict replication (same gene and same SNP) was not possible in our sample since rs2333227 was not present in the GWAS database, thus not evaluated in this study and, as found by Wenten *et al*., rs10001179 alone was not associated with asthma in our study (*p* = 0.942). Gene-gene interactions were evaluated between these two genes (*CAT* and *MPO*) to look at possible epistatic effects between other SNPs of these genes and asthma in a context of air pollution, but no association was found (results not shown). The haplotype block 2 of the *CAT* gene is also associated with asthma in this sample. This haplotype block is more transmitted to asthmatic subjects compared to controls and appears to be a susceptibility factor for asthma. Interestingly, this haplotype block includes the only SNP associated after correction (rs11032703), for which the minor allele is also transmitted more often to the asthmatic subjects. However, it is important to note that the associated SNPs may not be the causal one and other SNPs in linkage disequilibrium with the associated one may explain the impact on the trait.

These results indicate the importance of this locus and of the *CAT* gene in asthma. The fact that SNPs located in the *CAT* gene were not associated after correction when the sample was stratified according to the proximity of aluminium industries and that no association was observed when the sample was stratified according to the proximity of pulp and paper industries may indicate that the association of this gene is not restricted to a specific environment. As previously mentioned, the origin of the causal SNP may not be the one associated in this study and its impact on asthma needs to be assessed. However, since the exposition measure is not perfect, this clearly points out the importance of measuring and modeling individual-level exposures to air pollutants in order to properly study gene-environment interactions so that firm conclusions can be drawn. 

## 5. Conclusions

In conclusion, in this study we evaluated the association of genes that were previously associated with asthma in the literature within a gene-environment interaction with air pollution context. *CAT* gene previously associated with urban environments and air pollution was associated with asthma after correction in our sample. Interestingly, the familial collection used in this study is characterized by being composed of a remote/rural population. However, subjects tend to live in an area with variable, but potentially significant air pollutant exposures because there are aluminium, pulp and paper, and wood product industries in the region which emit a range of pollutants like SO_2_ and particulate matter. However, while these are important pollution sources, the surrounding background pollutant levels are generally low. This differs from the environment in urban areas where there are many sources (e.g., traffic) leading to higher overall levels of air pollutants, but which are less variable and potentially with less intense peaks typically associated with industrial plumes. In order to determine if the genes found to be important in this study were indeed implicated because of environmental (air pollutant) exposures and not because of other unknown factors, future efforts need to focus on accurate characterization of individual exposures from all potential sources. This added information would allow for a more complete assessment of the impact of gene-environment interactions in asthma and could lead to better insight regarding what types and quantities of exposures are harmful with respect to different asthma phenotypes. Finally, this study gives first exploratory results of gene-environment interactions in this area and shows that this kind of study is important to better understand asthma pathogenesis. However, better characterization tools will be needed to correctly answer those questions. This study also emphasizes the challenge that these types of studies represent and raises some questions regarding a few points that need to be taken into consideration for future approaches: (1) How to measure the environment; (2) when to measure it (what period of life); (3) how long do we measure it; and (4) what environment (in this case what air pollutant) to consider.

## Supplementary Files

Supplementary File 1: PDF-Document (PDF, 378 KB)
