# Supplementary material for: Association Study of Genes Associated to Asthma in a Specific Environment, in an Asthma Familial Collection Located in a Rural Area Influenced by Different Industries
Source: Int J Environ Res Public Health. 2012 Jul 27;9(8):2620–35. doi: 10.3390/ijerph9082620 (PMC3447577; doi:10.3390/ijerph9082620)
Supplement: Supplementary File 1: — PDF-Document (PDF, 378 KB) [file ijerph-09-02620-s001.pdf]

OPEN ACCESS

International Journal of
Environmental Research and Public Health
ISSN 1660-4601
www.mdpi.com/journal/ijerph

Supplementary Information

Association Study of Genes Associated to Asthma in a Specific Environment, in an Asthma Familial Collection Located in a Rural Area Influenced by Different Industries. *Int. J. Environ. Res. Public Health* 2012, *9*, 2620–2635

Andréanne Morin [1], Jeffrey R. Brook [2], Caroline Duchaine [3] and Catherine Laprise [1,4,*]

[1] Université du Québec à Chicoutimi, 555 boulevard de l'Université, Saguenay, QC G7H 2B1, Canada; E-Mail: andreanne1_morin@uqac.ca

[2] Air Quality Processes Research Section, Environment Canada Dalla Lana School of Public Health, University of Toronto, 4905 Dufferin St., Toronto, ON M3H 5T4, Canada; E-Mail: Jeff.Brook@ec.gc.ca

[3] Institut Universitaire de Cardiologie et Pneumologie de Québec, Hôpital Laval 2725, Chemin Sainte-Foy, QC G1V 4G5, Canada; E-Mail: Caroline.Duchaine@bcm.ulaval.ca

[4] Community Genomic Medicine Centre, University of Montréal, Chicoutimi University Hospital, 305, Rue Saint-Vallier, C.P. 5006, Saguenay, QC G7H 5H6, Canada

* Author to whom correspondence should be addressed; E-Mail: Catherine.Laprise@uqac.ca; Tel.: +1-418-545-5011 (ext. 5659); Fax: +1-418-615-1203.

Received: 30 April 2012; in revised form: 22 June 2012 / Accepted: 10 July 2012 / Published: 27 July 2012

Table 1. Polymorphisms analyzed in this study.

Gene/ Location	SNP [a]	Major/minor allele	HWE Total	HWE Alu.	HWE P.P.	MAF Total	MAF Alu.	MAF P.P.
EPHX1/ 1q42.1	rs4653436	G/A	0.1965	0.2078	0.1123	0.388	0.39	0.418
	rs1467143	A/G	0.3497	0.4249	1.0	0.465	0.458	0.459
	rs2854451	G/A	0.3441	0.3754	0.1059	0.251	0.251	0.259
	rs3753658	G/T	0.7496	0.7854	1.0	0.092	0.09	0.092
	rs3753659	T/C	0.7524	0.7854	1.0	0.093	0.09	0.092
	rs3753660	T/C	0.8633	0.9045	1.0	0.096	0.094	0.094
	rs3738040	G/A	0.3601	0.2904	0.3594	0.09	0.085	0.081
	rs1877724	C/T	0.318	0.3239	0.1325	0.252	0.253	0.261
	rs3738042	G/A	0.3519	0.4105	0.2769	0.381	0.382	0.411
	rs3753661	T/G	0.4392	0.5556	0.4616	0.098	0.093	0.085
	rs3766934	G/T	0.4345	0.5499	0.4616	0.097	0.093	0.085
	rs2671272	G/A	0.1761	0.3688	0.2659	0.177	0.183	0.153
	rs3738043	A/G	0.4345	0.5499	0.4616	0.097	0.093	0.085
	rs2854456	C/T	0.3123	0.3173	0.1325	0.252	0.252	0.261
	rs4149222	G/T	1.0	1.0	1.0	0.096	0.093	0.097
	rs2671270	A/G	0.4024	0.2629	0.1531	0.248	0.248	0.253
	rs2234698	T/C	0.5481	0.1424	0.4801	0.058	0.051	0.052
	rs2292566	G/A	N/A	0.0578	0.9592	N/A	0.138	0.138
	rs2740170	C/T	0.9511	0.6142	0.3504	0.258	0.258	0.261
	rs4149223	G/C	0.5799	0.5367	0.94	0.47	0.459	0.463
	rs2234922	A/G	0.1111	0.3494	0.1821	0.15	0.147	0.146
	rs4149226	C/T	0.4258	0.4352	0.906	0.426	0.429	0.428
	rs4653695	A/C	0.8173	0.8355	0.4808	0.119	0.128	0.119
	rs2740174	A/G	0.8649	1.0	0.8001	0.07	0.069	0.075
	rs360063	G/A	0.307	0.5275	0.7847	0.42	0.428	0.418
	rs1009668	C/T	0.9463	0.9118	0.4852	0.116	0.126	0.118
	rs868966	A/G	N/A	N/A	0.3135	N/A	N/A	0.385
	rs1051740	T/C	N/A	N/A	0.1685	N/A	N/A	0.237
ARG1/ 6q23	rs2608897	C/T	N/A	0.7752	0.5588	N/A	0.281	0.28
	rs2246012	T/C	0.211	0.3927	1.0	0.124	0.127	0.131
	rs17184300	T/C	0.3608	0.6766	0.1015	0.134	0.128	0.128
TNF/ 6p21.*3*	rs5029936	G/A	1.0	1.0	1.0	0.086	0.09	0.253
	rs643177	C/T	0.5413	1.0	0.4012	0.233	0.233	0.331
	rs610604	T/G	0.2385	0.3479	0.4781	0.321	0.325	0.075

Table 1. *Cont.*

Gene/ Location	SNP [a]	Major/minor allele	HWE Total	HWE Alu.	HWE P.P.	MAF Total	MAF Alu.	MAF P.P.
CAT/ 11p13	rs208679	A/G	0.776	0.3975	0.9829	0.088	0.092	0.087
	rs7118388	A/G	0.4739	0.9746	0.41	0.468	0.47	0.455
	rs7944397	A/G	0.2007	0.6099	0.7369	0.156	0.154	0.139
	rs208682	C/T	0.9714	1.0	0.3155	0.164	0.162	0.16
	rs554518	C/T	0.7819	0.7891	0.2261	0.148	0.146	0.143
	rs1001179	C/T	0.1644	0.134	0.1824	0.184	0.183	0.168
	rs480575	A/G	0.1459	0.2977	0.7437	0.351	0.353	0.339
	rs2284369	A/G	0.1951	0.6386	0.7931	0.263	0.262	0.252
	rs11032703	C/T	0.7928	1.0	0.8758	0.124	0.129	0.143
	rs769218	G/A	0.2517	0.8278	0.834	0.261	0.259	0.251
	rs2300181	C/T	0.9497	0.9925	0.8759	0.206	0.198	0.225
	rs7933285	C/T	0.9497	0.9925	0.8759	0.206	0.198	0.225
	rs511895	C/T	0.5477	0.8572	0.8708	0.363	0.371	0.364
	rs10488736	C/T	0.3862	0.3464	0.8308	0.287	0.277	0.297
	rs1535721	G/A	0.8788	1.0	0.9272	0.295	0.298	0.275
	rs1323690	G/A	N/A	0.5708	0.0669	N/A	0.323	0.353
GSTP1/ 11q13	rs614080	A/G	0.7474	1.0	N/A	0.462	0.472	N/A
	rs6591256	A/G	0.6588	0.6878	0.1381	0.37	0.382	0.362
	rs4147581	G/C	0.7474	1.0	N/A	0.462	0.472	N/A
	rs1695	A/G	0.4381	0.2402	0.7368	0.324	0.337	0.325
	rs1138272	C/T	1.0	1.0	0.4452	0.069	0.068	0.064
NQO1/ 16q22.1	rs10517	G/A	N/A	0.5704	0.0588	N/A	0.17	0.159
	rs1800566	G/A	0.7716	0.7252	0.136	0.17	0.18	0.183
	rs4986998	G/A	0.6167	0.9934	0.7259	0.067	0.066	0.053
	rs689452	G/C	N/A	0.3336	0.0527	N/A	0.163	0.15
	rs1437135	A/G	0.937	0.9007	0.2439	0.171	0.181	0.184
	rs689457	C/T	0.0956	N/A	N/A	0.121	N/A	N/A
	rs2917682	T/C	N/A	0.2536	0.0973	0.178	0.174	0.156
MPO/ 17q23.1	rs8178375	T/C	0.4729	0.5018	0.8103	0.117	0.116	0.132
	rs2071409	T/G	0.3946	0.57	0.3958	0.141	0.145	0.164
	rs4401102	C/T	0.0714	0.0596	0.1895	0.217	0.223	0.232
	rs12452417	G/A	0.3009	0.2964	0.7865	0.138	0.137	0.144
TGFB1/ 19q13.1	rs10417924	C/T	0.7842	1.0	1.0	0.205	0.201	0.189
	rs8179181	G/A	0.6858	0.4754	0.1251	0.247	0.239	0.229
	rs8110090	A/G	1.0	1.0	0.6686	0.062	0.069	0.077
	rs4803455	A/C	0.7895	0.5588	0.5723	0.497	0.496	0.493
	rs2241715	C/A	0.4773	0.2594	0.5549	0.31	0.308	0.289
	rs2241714	C/T	0.3693	0.2023	0.2597	0.315	0.316	0.293

[a] 64 SNPs analyzed for all subjects, 69 SNPs for aluminum subjects and 69 SNPs for pulp and paper subjects; Definitions: SNP = single polymorphism nucleotide; HWE = Hardy-Weinberg equilibrium; MAF = minor allele frequency; Alu. = Aluminum industries; P.P. = pulp and paper industries; *EPHX1* = Epoxide Hydrolase 1 Microsomal; *ARG1* = Arginase; *TNF* = Tumor Necrosis Factor; *CAT* = Catalase; *GSTP1* = Glutathione S-Transferase pi 1; *NQO1* = NAD(P)H Dehydrogenase Quinone 1; *MPO* = Myeloperoxidase; *TGFB1* = Transforming Growth Factor, beta 1; N/A = not analyzed, did not respect the threshold for HWE, MAF or genotyping %.

Table 2. Proportions of individuals living within 10 km radius of different industries.

**Type of industry**	**Percentage (%) of individuals** [c]	**Type of pollutant produced by the industry** [d]
Aluminium	73.93	PAH, SO_2, fluoride, CO, $PM_{2.5}$, PM_{10}
Pulp and paper	44.59	VOC, $PM_{2.5}$, PM_{10}, SO_x, NO_x
Mine [a]	7.79	$PM_{2.5}$, PM_{10} [e]
Wood products	11.80	VOC, $PM_{2.5}$, PM_{10}, SO_x, CO
Others [b]	2.46	N/A

[a] Niobium mine; [b] Milk transformation, iron and steel, petroleum refining, chemicals, plastic and rubber, *etc.*; [c] These data were calculated with the NPRI (National pollutant release inventory) Google Earth software; [d] This information comes from Environment Canada [19,20] and NPRI Google Earth tool; [e] No data available for niobium mines; PAH = polycyclic aromatic carbon; SO_2 = sulfur dioxide; CO = carbon monoxide; PM = particulate matter; VOC = volatile organic compound; SO_x = sulfur oxides; NO_x= nitrogen oxides.

Table 3. Total pollutant emissions in tones from different types of industry located in the SLSJ area for the year 2002

**Pollutants**	**Aluminium**	**Pulp and paper**	**Wood product**	**Other industries**	**All facilities** [a]
Sulfur dioxide	25.854	1.727	N/A	N/A	1,978.139
Carbon monoxide	168.547	5.900	154	N/A	914.413
Volatile organic compound	169	585	379	15	256.189
PM-Total particulate matter	5.305	336	146	143	223.736
PM_{10}-Particulate matter; 10 microns	268.33	137	56	43	107.001
$PM_{2.5}$; 2.5 microns	217.04	116	41	4.3	58.575
Nitric oxides (NO_2)	1051	1147	244	N/A	579.648

Data were calculated using the NPRI Google Earth software; [a] All facilities reporting to the NPRI.

Figure S1. Pairwise linkage disequilibrium pattern of the *CAT* gene single nucleotide polymorphisms (SNPs). The location of each SNP on the chromosome is indicated on top. The numbers indicated in the diamonds correspond to the magnitudes of linkage disequilibrium (D') between the respective pairs of SNPs (for example, the pairwise magnitude of the linkage disequilibrium between rs554518 and rs480575 is 0.76). Diamonds in red without indicated number are in perfect linkage disequilibrium (D'= 1) and the ones that are in white are in equilibrium. Haplotype blocks are indicated by the black triangles. Block 2 is associated with asthma in the SLSJ familial collection.

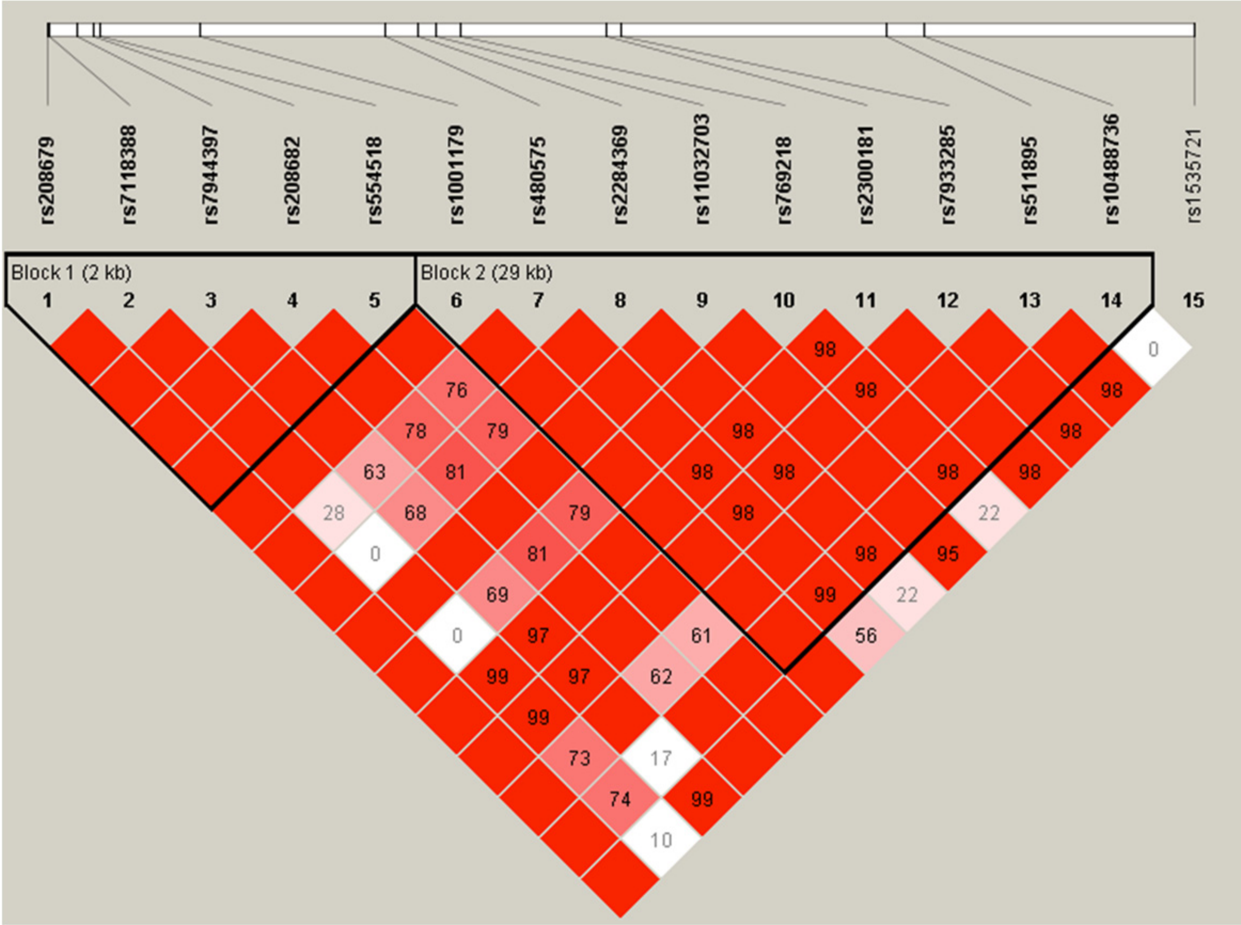

© 2012 by the authors; licensee MDPI, Basel, Switzerland. This article is an open access article distributed under the terms and conditions of the Creative Commons Attribution license (http://creativecommons.org/licenses/by/3.0/).
